# A green-electrospun nanofibrous scaffold incorporating polyethylenimine-modified liposomes for sustained BMP2 gene delivery and enhanced osteogenic differentiation

**DOI:** 10.3389/fbioe.2026.1748649

**Published:** 2026-02-04

**Authors:** Lin Zhang, Mengxia Chen, Zhen Chen, Sunfei Chen, Shenghan Duan, Shuqi Lou, Hongsheng Wang

**Affiliations:** 1 Department of Clinical Pharmacy, Shaoxing People’s Hospital, Shanghai, China; 2 Shanghai Engineering Research Center of Nano-Biomaterials and Regenerative Medicine, College of Biological Science and Medical Engineering, Donghua University, Shanghai, China; 3 Laboratory of Dental Biomaterials and Tissue Regeneration, Shanghai Xuhui District Stomatological Hospital, Shanghai, China

**Keywords:** green electrospinning, nanofiber, osteogenic induction, PEI-modified liposome, silk fibroin

## Abstract

**Introduction:**

Bone tissue engineering requires scaffolds that mimic the native extracellular matrix and provide sustained delivery of osteoinductive factors. This study focuses on developing a multifunctional scaffold using a green electrospinning process to combine the biocompatibility of silk fibroin (SF) with a non‐viral gene delivery system for sustained expression of Bone Morphogenetic Protein 2 (BMP2).

**Methods:**

A green electrospinning technique, using an aqueous SF and polyethylene oxide (PEO) solution, was employed to fabricate nanofibrous scaffolds, eliminating the use of harsh organic solvents. Polyethylenimine (PEI) modified liposomes (Lipo^PEI^) were used to encapsulate a BMP2‐encoding plasmid (pDNA_*Bmp2*_). These gene‐loaded nanoparticles were incorporated into the SF‐PEO nanofibers. The resulting scaffolds were characterized for morphology (SEM), structure (FTIR, XRD), and drug release kinetics. Biological performance was evaluated by assessing cell viability (MTT assay), cell attachment (SEM), gene transfection efficiency (confocal microscopy), and osteogenic differentiation (alkaline phosphatase (ALP) activity, Alizarin Red S staining) using bone marrow mesenchymal stem cells (BMSCs).

**Results:**

Physicochemical characterization confirmed the successful formation of uniform pDNA_Bmp2_@Lipo^PEI^ nanocomplexes with a particle size of approximately 266 nm and a positive surface charge of +16.9 mV. These nanocomplexes were homogeneously incorporated into smooth, bead-free SF‐PEO nanofibers with average diameters ranging from 460 to 541 nm. The composite scaffold demonstrated a highly sustained release of pDNA_Bmp2_ over 14 days. In vitro studies using rat bone marrow-derived mesenchymal stem cells (BMSCs) revealed that the scaffold possesses excellent biocompatibility, promoting robust cell adhesion, spreading, and proliferation. Furthermore, the gene‐loaded scaffold successfully mediated the transfection of BMSCs, leading to significant upregulation of osteogenic markers, including alkaline phosphatase (ALP) activity and extensive calcium mineral deposition over 21 days.

**Discussion:**

The novel composite scaffold combines the structural advantages of SF with a sustained BMP2 gene delivery system, showing remarkable potential to promote osteogenic differentiation. This work presents a promising, environmentally friendly, and effective platform for bone tissue engineering and regenerative medicine.

## 1 Introduction

The regeneration of bone tissue remains a significant clinical challenge in orthopedics, dentistry, and reconstructive surgery. Large bone defects resulting from trauma, tumor resection, infection, or congenital abnormalities often exceed the body’s intrinsic healing capacity, necessitating surgical intervention (Stevens, 2008; Dimitriou et al., 2011). While autografts are considered the clinical gold standard, their application is constrained by limited availability, donor site morbidity, and complications such as pain and infection (Younger and Chapman, 1989). Allografts and xenografts, on the other hand, present substantial risks of disease transmission and immunogenic rejection (Delloye et al., 2007). These limitations have catalyzed the development of bone tissue engineering, a multidisciplinary field that aims to create functional, synthetic bone substitutes capable of promoting effective and predictable bone regeneration (O’Brien, 2011).

A cornerstone of bone tissue engineering is the design of three-dimensional (3D) scaffolds that mimic the architecture and function of the natural extracellular matrix (ECM). The native bone ECM is a complex, hierarchical nanocomposite of collagen fibrils and hydroxyapatite crystals, providing both structural integrity and a supportive microenvironment for cellular activities (Rho et al., 1998). Among various fabrication techniques, electrospinning has emerged as a powerful platform for creating nanofibrous scaffolds that closely replicate the topographical features of the native ECM (Li et al., 2010). These scaffolds are characterized by high porosity, extensive surface area-to-volume ratio, and an interconnected pore network, which collectively facilitate cell adhesion, migration, proliferation, and differentiation (Huang et al., 2003; Pham et al., 2006). As highlighted in recent literature, innovations in electrospinning have focused on process optimization, the development of composite materials, and functionalization to enhance the biological performance of scaffolds (Huang et al., 2024; Chinnappan et al., 2022).

The choice of biomaterial is critical to the success of a tissue engineering scaffold. Silk fibroin (SF), a natural protein polymer derived from *Bombyx mori* cocoons, has garnered substantial interest due to its unique combination of properties. SF exhibits exceptional biocompatibility, minimal immunogenicity, and remarkable mechanical properties, including high tensile strength and tunable elasticity (Fan et al., 2025; Yang et al., 2024). Furthermore, its degradation products are non-toxic amino acids that can be safely metabolized by the body (Rockwood et al., 2011). Recent studies have demonstrated the versatility of SF in various forms, including films, hydrogels, and particularly, electrospun nanofibers for applications ranging from vascular and neural tissue engineering to skin regeneration (Ma et al., 2024). A significant advantage of SF is its processability in aqueous solutions, which enables “green” fabrication routes that avoid the use of cytotoxic organic solvents like hexafluoroisopropanol (HFIP) (Dai et al., 2024). This aqueous-based processing not only improves the environmental sustainability of the manufacturing process but, more importantly, preserves the bioactivity of incorporated therapeutic molecules (Lin et al., 2016).

To further enhance the osteoinductive potential of scaffolds, researchers have focused on incorporating bioactive signaling molecules. Bone Morphogenetic Protein 2 (BMP2) is one of the most potent osteoinductive growth factors known, playing a pivotal role in the differentiation of mesenchymal stem cells (MSCs) towards an osteoblastic lineage and promoting bone formation (Vanhatupa et al., 2015; Kim et al., 2008). However, the clinical application of recombinant human BMP2 is hampered by its short biological half-life, rapid enzymatic degradation, and the need for supraphysiological doses, which can lead to adverse side effects such as inflammation, ectopic bone formation, and high costs (Zara et al., 2011; Carragee et al., 2011).

Gene therapy offers a compelling alternative to direct protein delivery. By delivering DNA encoding for BMP2, the patient’s own cells can be transformed into bioreactors for the sustained, localized production of the therapeutic protein (Evans, 2012). This approach has the potential to achieve long-term therapeutic effects at physiological concentrations, thereby mitigating the risks and costs associated with bolus protein delivery (Kawai and Ohura, 2016). Non-viral gene vectors are preferred for their superior safety profile over viral vectors. Among these, cationic liposomes have been widely investigated for their ability to encapsulate and protect genetic material, their biocompatibility, and low immunogenicity (Al-Dosari and Gao, 2009). To enhance their transfection efficiency, liposomes are often combined with cationic polymers like polyethylenimine (PEI). PEI is renowned for its high transfection efficacy, attributed to its “proton sponge” effect, which facilitates endosomal escape (Boussif et al., 1995). Hybrid PEI-liposome nanoparticles leverage the high transfection efficiency of PEI while mitigating its inherent cytotoxicity through the liposomal component, creating a more favorable balance between efficacy and safety (Taheri et al., 2024; Shayestehfar et al., 2022). Recent advances in this area have focused on optimizing formulations for large-scale manufacturing and developing stimulus-responsive systems for controlled gene release (Williams-Fegredo et al., 2024).

The integration of such a non-viral gene delivery system within a biodegradable nanofibrous scaffold creates a “gene-activated matrix”—a sophisticated platform that provides both the necessary structural support for tissue ingrowth and the sustained genetic cues for osteogenic differentiation (Chew et al., 2011). The scaffold serves as a localized reservoir for the gene vectors, protecting them from degradation and facilitating their uptake by cells migrating into the scaffold (Petersen et al., 2010). This strategy has been shown to enhance and prolong gene expression compared to the direct injection of gene vectors alone.

This study aims to develop and evaluate a novel, fully green-fabricated composite nanofibrous scaffold for bone regeneration. A biocompatible and mechanically competent scaffold capable of gene delivery was developed by incorporating an optimized PEI modified liposome system (Lipo^PEI^) carrying the BMP2 encoding plasmid (pDNA_*Bmp2*_) into aqueous-based, electrospun SF-PEO nanofibers (named as pDNA_*Bmp2*_@Lipo^PEI^@SF-PEO). The physicochemical properties of the material were characterized, and its osteoinductive properties were also evaluated through *in vitro* experiments using bone marrow-derived MSCs (BMSCs) This research addresses the critical need for advanced, safe, and effective biomaterials for bone tissue engineering with a strong emphasis on clinical translatability and environmental sustainability.

## 2 Materials and methods

### 2.1 Materials

Soybean lecithin, cholesterol, octadecylamine and polyethylenimine (PEI, 25 kDa, branched) were purchased from Sigma-Aldrich (St. Louis, MO, USA). *Bombyx mori* silkworm cocoons were sourced locally. Poly (ethylene oxide) (PEO, Mw = 900 kDa) was purchased from Aladdin Industrial Corporation (Shanghai, China). The pIRES2-ZsGreen1-BMP2 plasmid, containing the gene for BMP2 and a green fluorescent protein (GFP) reporter, was constructed and preserved by our laboratory. Dulbecco’s Modified Eagle Medium (DMEM), fetal bovine serum (FBS), and penicillin-streptomycin were purchased from Gibco (Grand Island, NY, USA). 3-(4,5-dimethylthiazol-2-yl)-2,5-diphenyltetrazolium bromide (MTT), 3,3′-dioctadecyloxacarbocyanine perchlorate (DiO), Alizarin Red S (ARS), and the alkaline phosphatase (ALP) activity assay kit were obtained from Beyotime Biotechnology (Shanghai, China). All other chemical reagents were of analytical grade and used as received. Rat BMSCs present in this study were obtained from Cell Bank and Stem Cell Bank of Chinese Academy of Sciences.

### 2.2 Preparation and characterization of PEI-liposome/DNA nanoparticles

Cationic Lipo^PEI^ were prepared using a thin-film hydration method. Briefly, soybean lecithin (0.1 g), cholesterol (0.01 g), and Octadecylamine (0.004 g) were dissolved in ethanol in a round-bottom flask. The organic solvent was evaporated under reduced pressure to form a thin lipid film, which was then hydrated with 10 mL PEI aqueous solution (0.001 g/mL) and the Lipo^PEI^ suspension was obtained after stirring 30 min at room temperature. The suspension was sonicated and repeatedly extruded through a 100 nm polycarbonate membrane to obtain uniformly sized Lipo^PEI^.

For the preparation of DNA-loaded Lipo^PEI^, the plasmid pIRES2-ZsGreen1-BMP2 (pDNA_*Bmp2*_) was mixed with the Lipo^PEI^ suspension at various mass ratios (Lipo^PEI^: pDNA_*Bmp2*_ = 20 : 1, 40 : 1, and 60 : 1) and incubated at room temperature to allow for complex formation. The particle size, polydispersity index (PDI), and zeta potential of the nanoparticles were measured by Dynamic Light Scattering (DLS) using a Zetasizer Nano ZS (Malvern Instruments, UK). The morphology of the nanoparticles was observed by Transmission Electron Microscopy (TEM, JEM-2100, JEOL, Japan) after negative staining with 4% phosphotungstic acid. The encapsulation efficiency (EE) of the plasmid DNA was determined indirectly. The amount of unencapsulated DNA in the supernatant after centrifugation of the complex was quantified using a UV-Vis spectrophotometer. The EE was calculated using the formula: EE (%) = (Total DNA - Free DNA)/Total DNA × 100%.

### 2.3 Fabrication of nanofibrous scaffolds loaded with pDNA_*Bmp2*_@Lipo^PEI^

The silk fibroin (SF) solution was prepared as previously described. Briefly, cocoons were degummed, dissolved in 9 M LiBr solution, and dialyzed against deionized water to obtain a purified SF aqueous solution. For electrospinning, PEO was added to the SF solution at 10% of the SF’s mass to improve spinnability. The optimized pDNA_*Bmp2*_@Lipo^PEI^ complexes were then added to the SF/PEO solution at different concentrations (mass percentage of Lipo^PEI^ relative to SF was set at 0.2%, 0.4%, and 0.6% respectively, while the amount of pDNA_*Bmp2*_ was fixed to 60 μg) and gently mixed to form a homogeneous spinning dope (Table 1). The solution was loaded into a syringe and electrospun using a standard electrospinning setup. The process parameters were optimized and set as follows: applied voltage of 10–13 kV, a tip-to-collector distance of 15–20 cm, and a solution flow rate of 0.3–0.5 mL/h. The nanofibers were collected on a grounded aluminum foil. After spinning, the scaffolds were treated with 75% ethanol vapor for 24 h to induce a conformational transition to the water-insoluble β-sheet structure. Four groups of scaffolds were fabricated: SF-PEO, Lipo^PEI^@SF-PEO (empty vector), pDNA_*Bmp2*_@SF-PEO (scaffold with naked plasmid), and pDNA_*Bmp2*_@Lipo^PEI^@SF-PEO.

**TABLE 1:** Consist of different spinning solution.

Name of the composites	SF (g)	PEO (g)	pDNA_*Bmp2*_ (μg)	12 mg/mL Lipo^PEI^ (mL)	Deionized H_2_O (mL)
SF-PEO	0.6	0.06	—	—	3.0
pDNA_*Bmp2*_@Lipo^PEI^ (0.2%)@SF-PEO	0.6	0.06	60	0.1	2.9
pDNA_*Bmp2*_@Lipo^PEI^ (0.4%)@SF-PEO	0.6	0.06	60	0.2	2.8
pDNA_*Bmp2*_@Lipo^PEI^ (0.6%)@SF-PEO	0.6	0.06	60	0.3	2.7
pDNA_*Bmp2*_@SF-PEO	0.6	0.06	60	—	3.0

### 2.4 Characterization of nanofibrous scaffolds

The surface morphology and fiber diameter of the scaffolds were examined using Scanning Electron Microscopy (SEM, S-4800, Hitachi, Japan) after sputter-coating with gold. The distribution of the Lipo^PEI^ within the nanofibers was observed using a fluorescent microscope (DMi 8, Leica Microsystems Ltd., Wetzlar, Germany) by labeling the liposomes with DiO. The chemical structure and conformation of the SF within the nanofibers were analyzed by Fourier Transform Infrared Spectroscopy (FTIR, Nicolet 6700, Thermo Fisher Scientific, USA). The crystalline structure of the scaffolds was evaluated by X-ray Diffraction (XRD, D/Max-2550 PC, RIGAKU, Japan).

### 2.5 *In Vitro* drug release study

The release profile of the plasmid DNA from the scaffolds was evaluated *in vitro*. A certain mass of electrospun fibrous scaffolds (3 parallel samples in each group) was immersed in PBS buffer and placed in an oscillating shaker (temperature maintained at 37 °C and oscillation frequency at 100 rpm). Remove 1 mL of release solution every 24 h while supplementing with fresh equal volume of PBS. The absorbance value of the samples is measured by a UV spectrophotometer (NanoDrop 2000C) at 260 nm, and the average value of each group of samples is calculated according to the following formula, and the sustained-release curve can be obtained with the percentage of each release to the actual drug load as the ordinate and the sustained-release time as the abscissa.

$$E_{r}=\frac{V_{e}\sum_{i}^{n-1}C_{i}+V_{0}C_{n}}{m_{drug}}$$

(Er: cumulative DNA release, %; V_e_: Displacement volume of extended-release solution, 1,000 μL; V_0_: Volume of release solution (μL); C_i_: Concentration of DNA in the release solution at the time of the first displacement sampling (ng/μL); m_drug_: total mass of DNA used for release (ng); n: Number of times the sustained-release solution was replaced).

### 2.6 Cell culture

Rat BMSCs were used for all cellular experiments. The cells were cultured in DMEM-F12 supplemented with 10% FBS and 1% penicillin-streptomycin in a humidified incubator at 37 °C with 5% CO^2^. The culture medium was changed every 2–3 days. Cells from passages three to five were used for the experiments.

### 2.7 *In Vitro* biocompatibility assessment

The biocompatibility of the scaffolds was assessed using the MTT assay. BMSCs were seeded onto the different scaffolds placed in a 24-well plate at a density of 5 × 10^4^ cells/well. Cells cultured on glass coverslips served as a control. After 1, 3, 5, and 7 days of culture, the MTT reagent was added to each well and incubated. The resulting formazan crystals were dissolved in DMSO, and the absorbance was measured at 492 nm using a microplate reader (MK3, Thermo, USA). Cell attachment and morphology on the scaffolds were observed by SEM after 3 days of culture. The cell-scaffold constructs were fixed, dehydrated, and prepared for SEM imaging.

### 2.8 Gene transfection efficiency

To evaluate the gene transfection efficiency, BMSCs were cultured on the pDNA_*Bmp2*_@Lipo^PEI^@SF-PEO scaffolds containing the GFP reporter gene. After 3 days of culture, the expression of GFP in the cells was observed using CLSM. Cells cultured on glass with free pDNA_*Bmp2*_@Lipo^PEI^ complexes served as a positive control. The percentage of GFP-positive cells was estimated to determine the transfection efficiency.

### 2.9 *In Vitro* osteogenic differentiation study

BMSCs were seeded on the scaffolds and cultured in an osteogenic induction medium. The osteogenic potential was evaluated by measuring ALP activity and calcium deposition.

ALP Activity Assay: After 7, 14, and 21 days of culture, the cells on the scaffolds were lysed with Triton X-100. The ALP activity in the cell lysate was measured using an ALP activity assay kit according to the manufacturer’s instructions. The total protein content was quantified using a BCA protein assay kit, and the ALP activity was normalized to the total protein content.

Alizarin Red S (ARS) Staining: After 14 and 21 days of culture, the cell-scaffold constructs were fixed and stained with 2% ARS solution (pH 4.1–4.3) for 30 min to visualize calcium deposits. For quantitative analysis, the stained calcium nodules were destained with 10% cetylpyridinium chloride, and the absorbance of the extracted solution was measured at 570 nm.

### 2.10 Statistical analysis

All quantitative data were presented as mean ± standard deviation (SD) from at least three independent experiments (n = 3). Statistical analysis was performed using one-way analysis of variance (ANOVA) followed by Tukey’s *post hoc* test for multiple comparisons. A *p*-value of less than 0.05 was considered statistically significant (*p < 0.05).

## 3 Results

### 3.1 Characterization of DNA-loaded Lipo^PEI^ nanoparticles

**FIGURE 1:**
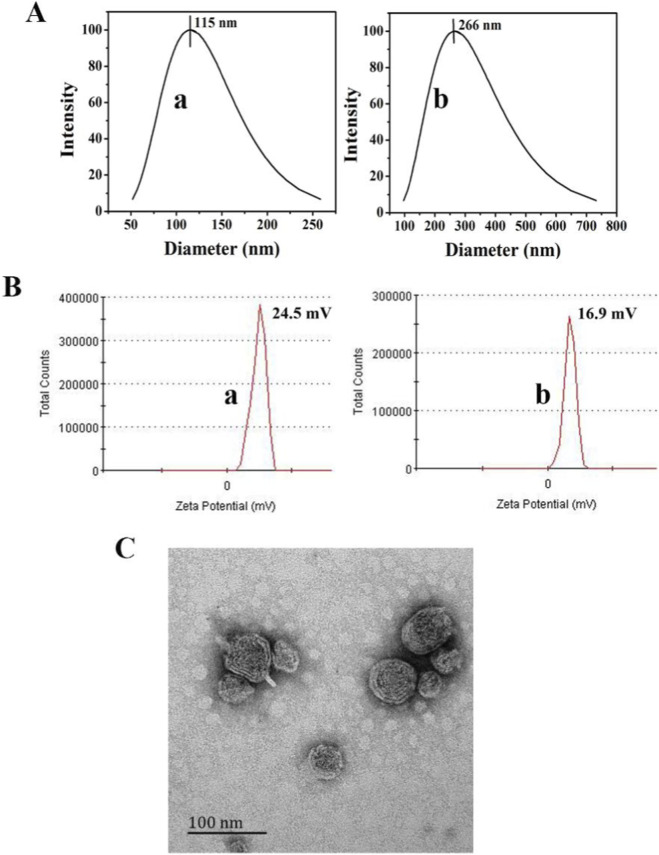
Particle size **(A)**, Zeta potential **(B)** and TEM photograph **(C)** of the nanocarriers ((a) Lipo^PEI^; (b) pDNA@Lipo^PEI^).

The physicochemical properties of the Lipo^PEI^ are crucial for successful gene delivery. As determined by DLS, the empty Lipo^PEI^ exhibited an average particle size of 115 ± 5 nm with a low PDI of 0.18 ± 0.02, indicating a uniform and monodisperse distribution (Figure 1A). The surface charge was strongly positive, with a zeta potential of +24.5 ± 2.1 mV, which is advantageous for binding negatively charged DNA (Figure 1B). Upon complexing with the pDNA_*Bmp2*_ at the optimized mass ratio of 40:1, the resulting pDNA_*Bmp2*_@Lipo^PEI^ nanoparticles showed an increase in average size to 266 ± 12 nm (Figure 1A), while the PDI remained low at 0.22 ± 0.03. The zeta potential decreased to +16.0 ± 1.8 mV (Figure 1B), confirming the successful binding of the anionic plasmid DNA to the cationic carrier surface. TEM imaging revealed that the nanoparticles were spherical and well-dispersed (Figure 1C). The encapsulation efficiency was calculated to be 66.23% ± 1.67%, demonstrating a competent capacity for gene loading.

### 3.2 Characterization of the nanofibrous scaffolds

**FIGURE 2:**
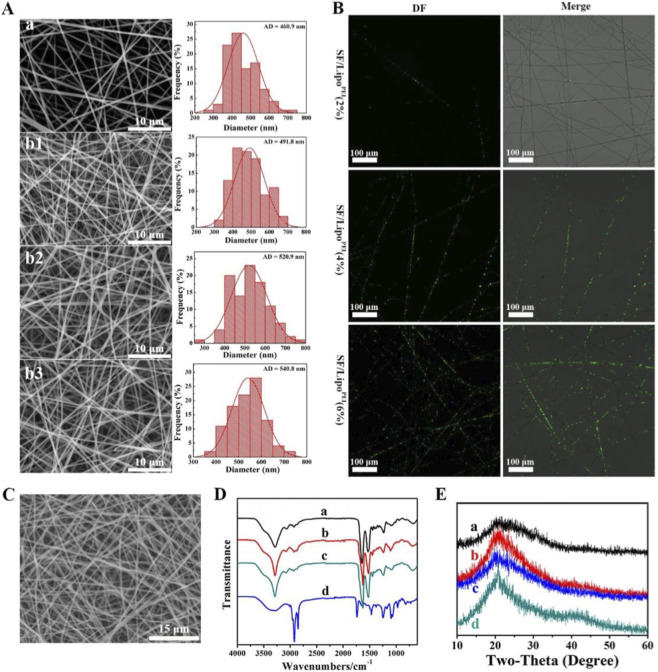
Characterization of the physichemical properties of pDNA_*Bmp2*_@Lipo^PEI^@SF Composite nanofibrous scaffolds. **(A)** SEM photographs and fiber diameter distribution analysis (b1: pDNA_*Bmp2*_@Lipo^PEI^(0.2%)@SF-PEO; b2: pDNA_*Bmp2*_@Lipo^PEI^(0.4%)@SF-PEO; b3: pDNA_*Bmp2*_@Lipo^PEI^(0.6%)@SF-PEO); **(B)** Fluorescence micrographs showing the presence and distribution of DiO-labeled Lipo^PEI^ in the fibers; **(C)** SEM image of 75% ethanol steam-treated pDNA_*Bmp2*_@Lipo^PEI^@SF-PEO scaffolds soaked in PBS solution for 7 days; **(D)** FTIR spectra of the scaffolds treated with 75% ethanol vapor; **(E)** XRD spectra of the scaffolds treated with 75% ethanol steam; a: SF-PEO; b: pDNA_*Bmp2*_@Lipo^PEI^@SF-PEO; c: pDNA_*Bmp2*_@Lipo^PEI^@SF-PEO; d: Lipo^PEI^.

SEM analysis was performed to evaluate the morphology of the electrospun scaffolds. As shown in Figure 2, all scaffold groups displayed a continuous, smooth, and ribbon-like nanofibrous structure, mimicking the native ECM. The pure SF scaffold had an average fiber diameter of 460.9 ± 45.2 nm. The incorporation of pDNA_*Bmp2*_@Lipo^PEI^ resulted in a slight, concentration-dependent increase in fiber diameter, with the pDNA_*Bmp2*_@Lipo^PEI^ (0.6%)@SF-PEO group reaching a diameter of 540.8 ± 55.3 nm (Figure 2A). This increase suggests the successful encapsulation of the nanoparticles within the fibers without disrupting the overall nanofibrous architecture. Microscopy of DiO-labeled liposomes confirmed their uniform distribution throughout the fiber matrix (Figure 2B). These composite nanofibrous scaffolds were treated with 75% ethanol vapor to obtain good hydration resistance and maintained their intact fiber structure after 7 days of soaking in PBS (Figure 2C). The structural changes in the SF protein upon ethanol treatment were assessed by FTIR and XRD. The FTIR spectrum of the as-spun fibers showed characteristic peaks for Amide I at 1,652 cm^-1^ (random coil), which shifted to 1,627 cm^-1^ (β-sheet) after ethanol treatment (Figure 2D). Correspondingly, the Amide II peak shifted from 1,533 cm^-1^ to 1,525 cm^-1^ (Figure 2D). These results confirm a successful conformational transition from a soluble random coil/α-helix state to a water-insoluble, stable β-sheet structure. XRD analysis supported this finding, showing a broad amorphous peak in the as-spun fibers that transformed into a sharper, more defined peak indicative of increased crystallinity after treatment (Figure 2E).

### 3.3 *In Vitro* plasmid DNA release

**FIGURE 3:**
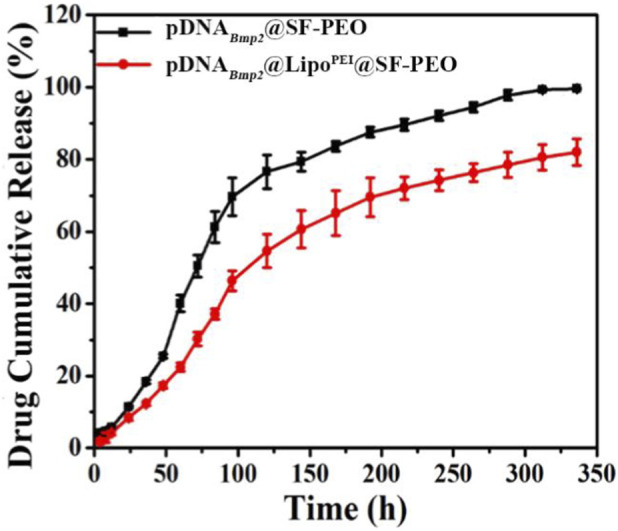
DNA release curve from the fibrous scaffolds.

The release kinetics of the pDNA_*Bmp2*_ from the scaffolds were monitored over 2 weeks. As shown in Figure 3, DNA release from the nanofibrous scaffolds with a rapid rate within the first 100 h and then gradually flattening. Obviously, the free DNA showed a faster release rate, and was almost fully released in 2 weeks. In contrast, the DNA encapsulated by Lipo^PEI^ was released more slowly, with only about 80% released in 2 weeks. This may be due to the adsorption of DNA by Lipo^PEI^, which delays the release of DNA, resulting in a long-term sustained release. The sustained release pattern is highly desirable for long-term gene expression and tissue regeneration.

### 3.4 Biocompatibility and cell morphology

**FIGURE 4:**
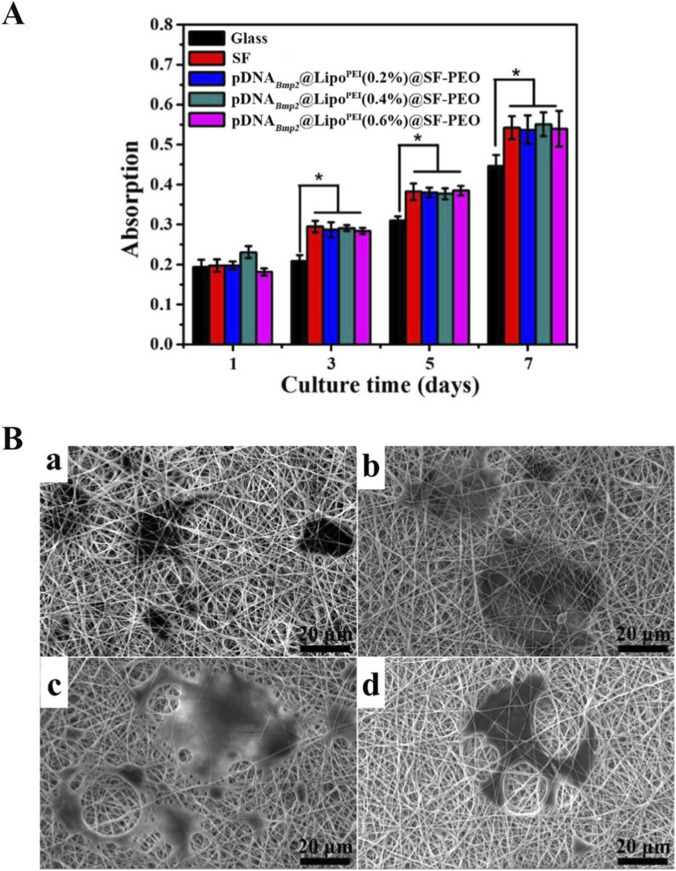
Proliferation and morphology of BMSCs cultured on the composite nanofibrous scaffolds. **(A)** cells viability data; (B) SEM image of the cells after 3 days of growth (B) ((a): SF-PEO; (b) Lipo^PEI^@SF-PEO; (c) pDNA_*Bmp2*_@Lipo^PEI^@SF-PEO; (d) pDNA_*Bmp2*_@SF-PEO).

The cytocompatibility of the scaffolds is essential for their application in tissue engineering. As shown in Figure 4A, BMSCs cultured on all scaffold groups exhibited robust proliferation over 7 days. Notably, the proliferation on the composite nanofibrous scaffolds was significantly higher than on the glass control after 3 days (*p* < 0.05), indicating that the scaffold materials and the incorporated gene vectors possess excellent biocompatibility and are conducive to cell growth. SEM imaging after 3 days of culture confirmed these findings. BMSCs were observed to adhere and spread well on the surfaces of all nanofibrous scaffolds. The cells displayed a healthy, flattened polygonal morphology with numerous filopodia extensions, indicating strong cell-matrix interactions (Figure 3B). The 3D porous structure of the scaffolds allowed for some cells to begin migrating into the scaffold interior, a promising sign for eventual tissue ingrowth.

### 3.5 Gene transfection efficiency

**FIGURE 5:**
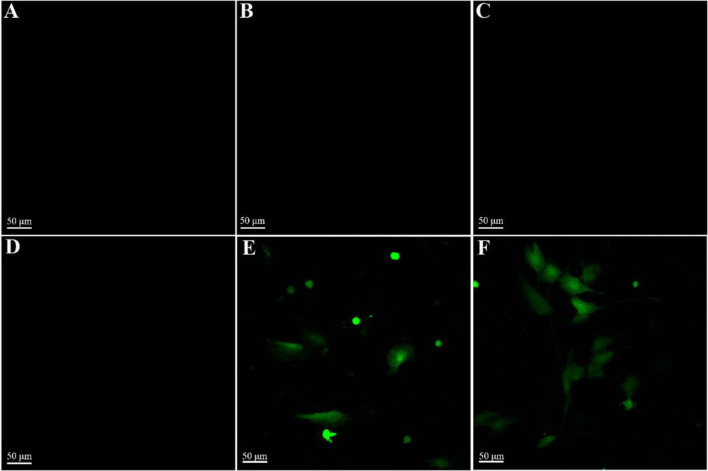
Confocal images of BMSCs cells growing for 7 days under different conditions (**(A)**: glass; **(B)** SF-PEO; **(C)** glass + pDNA_*Bmp2*_; **(D)** Lipo^PEI^@SF-PEO; **(E)** pDNA_*Bmp2*_@Lipo^PEI^@SF-PEO; **(F)** glass + pDNA_*Bmp2*_@Lipo^PEI^).

The ability of the gene-activated scaffolds to transfect BMSCs was evaluated by observing the expression of the GFP reporter gene. After 7 days of culture on the pDNA_*Bmp2*_@Lipo^PEI^@SF-PEO scaffold, strong green fluorescence was observed in cells via confocal microscopy (Figure 5E), which was comparable to the positive control where cells were cultured on glass and treated with free pDNA_*Bmp2*_@Lipo^PEI^ (Figure 5F). In contrast, no fluorescence was detected in the negative control groups (cells cultured on scaffolds with no pDNA_*Bmp2*_ or with naked pDNA_*Bmp2*_), confirming that the Lipo^PEI^ was essential for successful gene delivery and expression.

### 3.6 *In Vitro* osteogenic evaluation

To assess the ultimate biological function of the gene-activated scaffold, its ability to induce osteogenic differentiation of BMSCs was evaluated.

**FIGURE 6:**
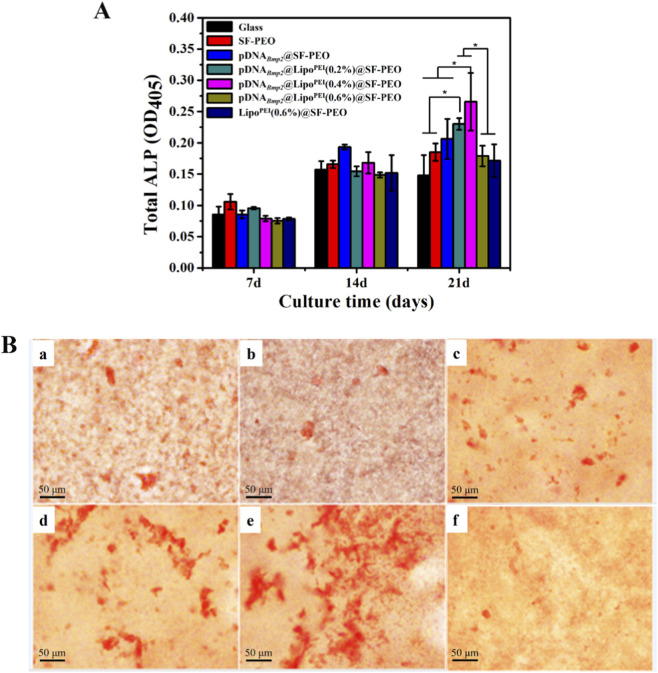
Expression of ALP in BMSCs grown on different scaffolds **(A)** and Alizarin red staining of the cells (B) ((a): SF-PEO; (b) Lipo^PEI^@SF-PEO; (c) pDNA_*Bmp2*_@Lipo^PEI^ (0.2%)@SF-PEO; (d) pDNA_*Bmp2*_@Lipo^PEI^ (0.4%)@SF-PEO; (e) pDNA_*Bmp2*_@Lipo^PEI^ (0.6%)@SF-PEO; (f) pDNA_*Bmp2*_@SF-PEO; *p < 0.05).

ALP Activity: ALP is an early marker of osteoblastic differentiation. As shown in Figure 6A, all groups supported a basal level of ALP activity. However, the pDNA_*Bmp2*_@Lipo^PEI^@SF-PEO group exhibited a significantly higher ALP activity at day 21 compared to all control groups (glass, SF-PEO, Lipo^PEI^@SF-PEO, glass + pDNA_*Bmp2*_). The ALP activity in the gene-activated scaffold group was significantly higher than that of the pure SF scaffold and other control groups (*p* < 0.05), demonstrating a potent and sustained osteoinductive effect driven by the expression of BMP2.

Calcium Deposition: Alizarin Red S staining was used to evaluate calcium mineralization, a hallmark of late-stage osteogenic differentiation. After 21 days, intense red staining was observed in the pDNA_*Bmp2*_@Lipo^PEI^@SF-PEO groups, indicating extensive mineralized nodule formation. This result strongly corroborates the ALP activity data and confirms that the sustained delivery and expression of the BMP2 gene from the green-electrospun scaffold effectively promotes the complete osteogenic differentiation of BMSCs.

## 4 Discussion

This study successfully demonstrated the design, fabrication, and *in vitro* validation of a novel gene-activated scaffold for bone tissue engineering, based on a completely green electrospinning process. The results confirm our hypothesis that incorporating a PEI-liposome-based BMP2 gene delivery system into an aqueous-derived silk fibroin nanofibrous matrix creates a biocompatible platform capable of supporting cell growth and promoting robust, sustained osteogenic differentiation of BMSCs. The significance of these findings lies in the integration of green chemistry principles with advanced gene delivery strategies to address key challenges in regenerative medicine.

A major innovation of this work is the utilization of a green electrospinning process. Conventional electrospinning of silk fibroin often necessitates the use of harsh and cytotoxic organic solvents like HFIP, which can denature delicate bioactive molecules and pose environmental and regulatory hurdles (Lin et al., 2016). Our aqueous SF-PEO system circumvents these issues entirely. The successful fabrication of morphologically sound nanofibers confirms the feasibility of this green approach. More importantly, this gentle processing environment was crucial for preserving the structural integrity and transfection capability of the incorporated DNA@Lipo^PEI^ nanoparticles. This aligns with the growing trend towards sustainable and biologically-friendly manufacturing in tissue engineering, as highlighted in recent reviews (Huang et al., 2024; Dai et al., 2024).

The choice and design of the gene delivery vector were critical to the scaffold’s function. Our results showed that the hybrid Lipo^PEI^ system provided a favorable balance of properties. The nanoparticles had a net positive charge and a size (∼266 nm) suitable for cellular uptake via endocytosis (Taheri et al., 2024). While the encapsulation efficiency of ∼66% suggests room for optimization, it was sufficient to load a therapeutic dose of the BMP2 gene. The most significant advantage conferred by the carrier system, in concert with the scaffold matrix, was the sustained release profile. Unlike the burst release from scaffolds with naked DNA, the dual encapsulation (within the liposome and then the fiber) slowed the diffusion of the plasmid, providing a controlled release over at least 14 days. This temporal control is critical for mimicking the natural progression of bone healing and ensuring prolonged BMP2 expression, a key advantage over the short half-life of directly delivered recombinant BMP2 protein (Zara et al., 2011; Kawai and Ohura, 2016). Our findings are consistent with recent literature advocating for nanoparticle-based systems to achieve long-term gene expression in tissue engineering contexts (Williams-Fegredo et al., 2024).

The biological performance of the gene-loaded scaffold was outstanding. The excellent biocompatibility, demonstrated by high cell proliferation rates and favorable cell morphology, can be attributed to the inherent properties of silk fibroin (Fan et al., 2025; Yang et al., 2024). The nanofibrous architecture provided a high surface area for cell attachment, and the material itself is known to support osteogenic lineage progression. However, the most striking result was the potent induction of osteogenesis. The significant increase in ALP activity and calcium deposition by the BMP2-gene activated group are clear evidence of advanced osteogenic differentiation. This demonstrates that the transfected cells were successfully producing biologically active BMP2 protein, which then acted in an autocrine and paracrine manner to stimulate the differentiation cascade. This process involves the activation of the canonical Smad signaling pathway, leading to the upregulation of key osteogenic transcription factors and, ultimately, matrix mineralization (Vanhatupa et al., 2015; Kim et al., 2008), a mechanism well-documented in the BMP2 literature.

The study validates the concept of a multi-component, synergistic tissue engineering construct. The SF-PEO scaffold provided the essential 3D structural template for cell colonization. The Lipo^PEI^ served as an efficient vector for cellular transfection. The BMP2 gene provided the crucial biological instructions. The combination of these elements into a single gene-loaded scaffold created a system where the whole is greater than the sum of its parts. The scaffold localizes the gene therapy, the gene therapy functionalizes the scaffold, and the green process makes it all possible without compromising biological activity.

Despite the promising results, we acknowledge some limitations in this study. First, the entire evaluation was conducted *in vitro*. While these results are a crucial proof-of-concept, the ultimate efficacy of the scaffold must be validated in an *in vivo* animal model of a critical-sized bone defect. Second, the mechanical properties of the hydrated scaffolds were not quantitatively evaluated, comprehensive mechanical testing need to do to ensure the scaffold can provide adequate temporary support in a load-bearing environment. Finally, the degradation kinetics of the scaffold in a physiological environment require further investigation.

## 5 Conclusion

In this study, we have successfully developed and validated a multifunctional, gene-loaded nanofibrous scaffold using an entirely aqueous-based, green electrospinning process. The scaffold, composed of SF and PEO, effectively incorporated Lipo^PEI^ carrying BMP2-encoding plasmid. The resulting construct demonstrated excellent biocompatibility, supporting the adhesion and proliferation of BMSCs. The integrated gene delivery system provided sustained release of the plasmid and achieved high transfection efficiency, leading to the prolonged expression of the osteoinductive factor BMP2. Consequently, the gene-loaded scaffold significantly enhanced the osteogenic differentiation of BMSCs, as evidenced by increases in both ALP activity and matrix mineralization. This work pioneers a fully green approach to fabricating a sophisticated gene-loaded matrix, presenting a safe, environmentally friendly, and highly effective platform with significant potential for clinical applications in bone tissue engineering and regenerative medicine.

## Data Availability

The original contributions presented in the study are included in the article/supplementary material, further inquiries can be directed to the corresponding author.
